# Utilisation, Availability and Price Changes of Medicines and Protection Equipment for COVID-19 Among Selected Regions in India: Findings and Implications

**DOI:** 10.3389/fphar.2020.582154

**Published:** 2021-01-14

**Authors:** Mainul Haque, Santosh Kumar, Jaykaran Charan, Rohan Bhatt, Salequl Islam, Siddhartha Dutta, Jha Pallavi Abhayanand, Yesh Sharma, Israel Sefah, Amanj Kurdi, Janney Wale, Brian Godman

**Affiliations:** ^1^ Unit of Pharmacology, Faculty of Medicine and Defence Health, Universiti Pertahanan Nasional Malaysia (National Defence University of Malaysia), Kuala Lumpur, Malaysia; ^2^ Department of Periodontology and Implantology, Karnavati University, Gandhinagar, India; ^3^ Department of Pharmacology, All India Institute of Medical Sciences, Jodhpur, India; ^4^ Department of Pediatric Dentistry, Karnavati University, Gandhinagar, India; ^5^ Department of Microbiology, Jahangirnagar University, Savar, Bangladesh; ^6^ Department of Conservative Dentistry and Endodontics, Rajasthan University of Health Sciences, Jaipur, India; ^7^ Pharmacy Department, Ghana Health Service, Keta Municipal Hospital, Keta-Dzelukope, Ghana; ^8^ Pharmacy Practice Department, School of Pharmacy, University of Health and Allied Sciences, Volta Region, Ghana; ^9^ Strathclyde Institute of Pharmacy and Biomedical Sciences, University of Strathclyde, Glasgow, United Kingdom; ^10^ Department of Pharmacology, College of Pharmacy, Hawler Medical University, Erbil, Iraq; ^11^ Independent Consumer Advocate, Brunswick, VIC, Australia; ^12^ Division of Clinical Pharmacology, Karolinska Institute, Karolinska University Hospital Huddinge, Stockholm, Sweden; ^13^ School of Pharmacy, Sefako Makgatho Health Sciences University, Ga-Rankuwa, South Africa; ^14^ School of Pharmaceutical Sciences, Universiti Sains Malaysia, Penang, Malaysia

**Keywords:** community pharmacists, India, lower- and middle-income countries, medicines, protective equipment, price rises, shortages

## Abstract

**Background:** COVID-19 has already claimed a considerable number of lives worldwide. However, there are concerns with treatment recommendations given the extent of conflicting results with suggested treatments and misinformation, some of which has resulted in increased prices and shortages alongside increasing use and prices of personal protective equipment (PPE). This is a concern in countries such as India where there have been high patient co-payments and an appreciable number of families going into poverty when members become ill. However, balanced against pricing controls. Community pharmacists play a significant role in disease management in India, and this will remain. Consequently, there is a need to review prices and availability of pertinent medicines during the early stages of the COVID-19 pandemic in India to provide future direction.

**Objective:** Assess current utilisation and price changes as well as shortages of pertinent medicines and equipment during the early stages of the pandemic.

**Our Approach:** Multiple approach involving a review of treatments and ongoing activities across India to reduce the spread of the virus alongside questioning pharmacies in selected cities from early March to end May 2020.

**Our Activities:** 111 pharmacies took part, giving a response rate of 80%. Encouragingly, no change in utilisation of antimalarial medicines in 45% of pharmacies despite endorsements and for antibiotics in 57.7% of pharmacies, helped by increasing need for a prescription for dispensing. In addition, increased purchasing of PPE (over 98%). No price increases were seen for antimalarials and antibiotics in 83.8 and 91.9% of pharmacies respectively although shortages were seen for antimalarials in 70.3% of pharmacies, lower for antibiotics (9.9% of pharmacies). However, price increases were typically seen for PPE (over 90% of stores) as well as for analgesics (over 50% of pharmacies). Shortages were also seen for PPE (88.3%).

**Conclusion:** The pandemic has impacted on utilisation and prices of pertinent medicines and PPE in India but moderated by increased scrutiny. Key stakeholder groups can play a role with enhancing evidenced-based approaches and reducing inappropriate purchasing in the future.

## Introduction/Background

### Healthcare System in India and Role of Community Pharmacists

Until recently, healthcare in India has largely been funded through out-of-pocket payments, with payments relatively stagnant at between 69% to over 75% of total healthcare expenditure (Kastor and Mohanty, 2018; Selvaraj et al., 2018; Sood and Wagner, 2020). The cost of medicines accounted for an appreciable proportion of this at over 60% of total expenditure and over 70% of out-of-pocket expenditures (Selvaraj et al., 2018; Satheesh et al., 2019), which is similar to other lower- and middle-income countries (LMICs) (Cameron et al., 2009). These out-of-pocket expenses have potentially catastrophic consequences if family members become ill, exacerbated by an increase in non-communicable diseases (NCDs) in India in recent years. As a result, up to 39 million people are pushed into poverty each year in India due to healthcare payments (Garg and Karan, 2009; Kastor and Mohanty, 2018; Reddy, 2018; Selvaraj et al., 2018). This is beginning to change with ongoing reforms to provide health insurance coverage for up to 100 million families in poverty (Reddy, 2018; Sood and Wagner, 2020); however, this will take time in view of a number of challenges including patient enrollment, agreeing the future role of physicians, necessary strengthening of regulatory systems and increasing the percentage of gross domestic product (GDP) spent on health (Reddy, 2018; Editorial, 2019; Sood and Wagner, 2020). These reforms will also involve improving healthcare delivery in rural areas to address current inadequacies, for instance through the instigation of 150,000 health and wellness centers as healthcare facilities (Kasthuri, 2018; Sood and Wagner, 2020).

In view of the contribution of the costs of medicines to total healthcare costs in India, coupled with existing high co-payments for visiting physicians and funding medicines, community pharmacies have and will continue to play an appreciable role in managing patients in India (Abdulsalim et al., 2018; Daftary et al., 2019; Nafade et al., 2019; Abdulsalim et al., 2020; Sousa Pinto et al., 2020). Key reasons include avoiding paying physician fees, especially for milder symptoms, accessibility with long opening hours, and a lack of queues compared with ambulatory care clinics (Soumya et al., 2016; Mohathasim Billah and Venkatesan, 2017; Daftary et al., 2019). As a result, community pharmacists in India are the first point of healthcare professional contact for up to 40% of patients with tuberculosis (TB) symptoms and an appreciable number of patients continue to seek their advice after diagnosis (Daftary et al., 2019).

Overall, community pharmacists in India play a crucial role in optimizing the use of medicines and improving patient outcomes whilst seeking to prevent the misuse of medicines (Mohathasim Billah and Venkatesan, 2017; Abdulsalim et al., 2018). This is similar to their roles in other countries where they are often the first healthcare professional patients consult regarding their illness, and they are increasingly involved in the management of chronic and other diseases (Khanal et al., 2016; Markovic-Pekovic et al., 2017; Akutey et al., 2018; Sousa Pinto et al., 2020). The cost of medicines to patients in India have been helped in recent years by ongoing reforms since 2013 to fix prices for essential medicines as well as encourage the prescribing of generic medicines where possible (HealthWorld, 2016; BioVoice, 2018; Pavithra, 2019). However, concerns have been raised about the knowledge of pharmacists regarding pharmaceutical care, the actual extent of pharmaceutical care activities undertaken in practice, and the continued self-purchasing of antibiotics that exists despite legislation, which along with increased utilisation rates of antibiotics in recent years has led to increased antimicrobial resistance (AMR) (Satheesh et al., 2019; Nafade et al., 2019; Farooqui et al., 2018; pal Jeyamani et al., 2018).

Reforms are also needed in India regarding the management of NCDs, with NCDs now the leading cause of death in India. NCDs accounted for 60% of all deaths in India in 2014 alongside continued high rates of infectious diseases including TB and growing AMR rates (Farooqui et al., 2018; Kastor and Mohanty, 2018; Daftary et al., 2019). The growing burden of NCDs including cardiovascular disease (CVD), hypertension, and diabetes, coupled with the associated need for medicines, will appreciably increase healthcare costs unless addressed, alongside increasing expenditure on antimicrobials with increasing utilisation (Farooqui et al., 2018; India State-Level Disease Burden Initiative CVDC, 2018; India State-Level Disease Burden Initiative Diabetes C, 2018; Ramakrishnan et al., 2019; Vijayakumar et al., 2019).

It is likely therefore that any Government activities to transfer funds and personnel to tackle COVID-19 will impact on planned activities to reduce the growing burden of NCDs in India as well as any planned activities surrounding other infectious diseases including reducing AMR rates. Consequently, existing healthcare needs and activities need careful monitoring to reduce the extent any unintended consequences arising from the pandemic.

### COVID-19 and Risk Factors

COVID-19 was first identified Wuhan in China in December 2019, quickly spreading to all continents (Kumar et al., 2020; Li et al., 2020; Wu and McGoogan, 2020). By September 27, 2020 there were 32.73 million cases with over 990,000 deaths worldwide, giving a case fatality ratio (CFR) among confirmed cases of 3.03% (WHO, 2020a). This included over 6.72 million confirmed cases in the WHO South East Asian Region including India, with over 110,000 deaths, giving a CFR of 1.65% (WHO, 2020a). It is recognised that there has been appreciable under-reporting of prevalence rates and deaths in a number of South East Asian countries with a lack of testing capabilities, particularly at the beginning (Anwar et al., 2020; Associated Press, 2020; Lee, 2020).

COVID-19 is transmitted from person to person principally through respiratory droplets and aerosol transmission, alongside direct contact with contaminated surfaces, forming the basis of preventative measures (Anderson et al., 2020; Haque, 2020; Klompas et al., 2020; Ng et al., 2020; Pradhan et al., 2020; WHO, 2020b; World Health Organisation, 2020). Increased morbidity and mortality from COVID-19 appears to be associated with a number of underlying health conditions including CVD, hypertension, diabetes, chronic obstructive pulmonary disease (COPD), shortness of breath, smoking and blood types (Aghagoli et al., 2020; Alqahtani et al., 2020; Ellinghaus et al., 2020; Huang et al., 2020; Richardson et al., 2020; Vardavas and Nikitara, 2020; Zhao et al., 2020; Zheng et al., 2020). Ethnicity may also be important with patients in the United Kingdom of Indian origin at appreciably increased risk of dying from COVID-19 vs. those of white ethnicity (Khunti et al., 2020; Kirby, 2020; Public Health England, 2020; Sonwalkar, 2020). Smoking is also an issue in India with high rates particularly among men (Mishra et al., 2016; Mohan et al., 2018; WHO, 2018); however, rates appear to be decreasing following the instigation of the WHO’s framework convention on tobacco control and other measures, which is encouraging (Suliankatchi Abdulkader et al., 2019).

### Response to COVID-19 and Concerns in India

The first reported cases in India for COVID-19 were in Kerala, on January 30, 2020 (Kamath et al., 2020; Kumar et al., 2020; WHO India, 2020a). As of September 27, 2020, 5.992 million confirmed cases and over 94,000 deaths have been reported in India due to COVID-19, the highest rates in the WHO South East Asia region and giving a CFR of 1.58% (WHO, 2020a). This is likely to be an underestimate though with lack of testing and capacity issues certainly during the early stages of the pandemic until the number of testing facilities appreciably increased with the help of the private sector Box 1, exacerbated in some cases by patients having to cover the high costs of tests themselves although this is now changing (Alluri and Pathi, 2020; Associated Press, 2020). By late March, there were only 18 tests being undertaken per million population in India vs. 6,931 per million in South Korea (Kamath et al., 2020). By 25 April, over 5.79 million tests had been undertaken from data supplied by the Indian Council of Medical Research (ICMR) (Pai et al., 2020). Other reports suggest a cumulative total of 3.8 million tests by June 1, 2020 also citing ICMR. The testing rates at the end of May/beginning of June were still only 0.08 per 1,000 people in India vs. 1.02 in Italy and 1.16 in the USA (Yadav et al., 2020). Since then, with the help of an appreciable increase in the number of testing facilities and increased production of testing equipment (ICMR, 2020a; Yadav et al., 2020), the cumulative number of tests as of July 8, 2020 was over 110 million with 1,132 laboratories now involved with testing throughout India (ICMR, 2020b; ICMR, 2020c). ICMR has also recently approved the use of a point-of-care rapid antigen test to aid early detection of COVID-19 to further enhance the numbers tested to aid the “test-track-treat” strategy (Bhargava, 2020; ICMR, 2020d).

BOX 1 **Testing capabilities and type in India (Yadav et al., 2020; ICMR, 2020c).**

•Different methods are used to test for COVID-19. These include:
  ○ ICMR-validated PT-PCR (reverse transcription polymerase chain reaction test)
  ○ MolBio Diagnostics' Truenat (completely indigenous diagnostic platform (ICMR, 2020e)
•Facilities for the various testing approaches as of 31 May 2020:
  ○ Real-time RT-PCR: 481 facilities - government: 313 and private: 168
  ○ Truenat test for COVID-19: 140 facilities - government: 131 and private: 9
  ○ CBNAAT for COVID-19: 55 facilities - government: 28 and private: 27
•Facilities for the various testing approaches as of 8 July 2020:
  ○ Real-time RT-PCR: 603 facilities - government: 373 and private: 230
  ○ Truenat test for COVID-19: 435 facilities - government: 400 and private: 35
  ○ CBNAAT for COVID-19: 94 facilities - government: 33 and private: 61

India faces ongoing challenges with preventing the spread of COVID-19, including issues with social distancing in big cities with crowded streets, lack of access to clean water, lack of regular hand washing facilities in an appreciable number of households, lack of physicians, lack of hospital beds (at 0.5 to 0.7 beds per 1,000 compared with 4.3 per 1,000 in China) and ICUs vs. higher income countries, just 20,000 ventilators in the country, and a lack of personal protective equipment (PPE) among healthcare professionals (Bhattacharya et al., 2020; Dutta, 2020; Ganapathy, 2020; Garattini et al., 2020; Kamath et al., 2020; Ma and Vervoort, 2020; Roy et al., 2020; Saaliq, 2020).

There are also challenges with high co-payments for treating existing infectious and non-infectious diseases, which will be exacerbated by diverting expenditures towards prevention and management of COVID-19, as well as purchasing basic necessities if incomes are reduced as a result of the pandemic (Ganapathy, 2020; Kamath et al., 2020). This is in addition to concerns about the routine availability of medicines to treat priority diseases in India. Medicine prices are typically lower in India compared with a number of other LMICs although appreciable variation in prices has been seen among pharmacies (Millard et al., 2018; Babar et al., 2019; Ray et al., 2020). Having said this, as mentioned, there are ongoing activities by the Indian Government to regulate the prices of medicines in India to help with co-payments for essential medicines (HealthWorld, 2016; Pavithra, 2019). Purchasing personal protective equipment (PPE) remains an issue with the costs of the materials to make PPE appreciably increasing following the pandemic (Business Today, 2020); however, prices and shortages may be alleviated by increased local production along with pricing and manufacturing regulations (Bhattacharya et al., 2020; GMA Consulting Group, 2020; Pandey, 2020).

Ongoing national and regional activities during the early stages of the pandemic to help reduce the spread of COVID-19, as well as the financial consequences, are contained in Table 1. It was perceived that timely decisions to introduce lockdowns helped with controlling infection rates initially although at a negative socioeconomic cost (Pai et al., 2020). However, we are now seeing a spike in infection rates as lockdown measures are relaxed despite warnings to maintain social distancing and as a consequence of the recent monsoons (Associated Press, 2020; Siddiqui, 2020). Consequently, there has been a re-instigation of such measures although rail and other services have resumed and places of worship have opened (ANI, 2020; The Hindu, 2020).

**TABLE 1:** Range of national and regional activities in India in 2020 from January 2020 up to June 2020 to help reduce the spread of COVID 19 and its impact.

Activities	Details of activities including timelines during 2020 where documented
Healthcare activities	•January 30, 2020—Surveillance is being strengthened at points of entry into the country and in the community and the WHO issued advice to patients on hand sanitisation and other measures (WHO India, 2020a)
	•February 6, 2020—The government issued travel restrictions to China and anyone with a travel history from China from January 15, 2020 will be quarantined (WHO India, 2020b). The WHO is also working with key government and other groups to offer support and help (WHO India, 2020b). During this period, the ministry of health instigated a 24 h/7 days-a-week disease alert helpline to provide information including clinical guidance (Kumar et al., 2020; Ministry of Health and Family Welfare Government of India, 2020), with the alcohol industry engaged in developing hand sanitisers and the textile industry producing PPE to address shortages (Bhattacharya et al., 2020)
	•March 8, 2020—52 laboratories were identified by ICMR for undertaking testing for COVID-19 (WHO India, 2020c) (now expanded in Box 1). In addition, all international passengers irrespective of nationality are now mandated to undergo universal medical screening at airports with screening facilities in place at 30 airports and WHO India is supporting capacity building and training across India (WHO India, 2020c). Those arriving from China, France, Germany, Italy, Iran, Republic of Korea, Spain and Germany after February 15, 2020 to be quarantined for a minimum of 14 days starting March 13, 2020 (WHO India, 2020d)
	•March 15, 2020 - all movement suspended for foreigners through all immigration land check posts at Bangladesh, Nepal, Bhutan and Myanmar borders with only a few exceptions (nepalese and bhutanese nationals)
	•March 22, 2020: “Janata curfew” introduced with 14 hour lockdown (Kumar et al., 2020). In addition, all train services suspended until 31 March, although goods trains may continue for essential commodities, and all children under 10 and all elderly over 65 to remain at home and avoid mass gatherings unless a medical reason or for essential services (WHO India, 2020e)
	•Ministry of Pharma and Consumer Affairs instructed to take necessary action to regulate the price for PPE and other health related materials and to facilitate their availability in hospitals and to the population at large (WHO India, 2020e)
	•WHO working with ICMR to enhance the testing strategy (WHO India, 2020e) (now appreciably expanded—Box 1), with ICMR issuing guidelines for the clinical management of patients with COVID-19
	•March 25, 2020—Further lockdown measures initiated for 21 days starting on 25 March (Kamath et al., 2020; Kumar et al., 2020; PIBG, 2020), and extended to 3 May (Kumar et al., 2020).
	•India introduced an export ban on hydroxychloroquine but later rescinded as seen in for instance Malaysia (Duffy & Hussain, 2020; Sibbal, 2020)
	•April 2020—ICMR issued national guidelines for ethics committees reviewing biomedical and health research during the COVID-19 pandemic (Indian Council of Medical Research, 2020)
	•April 14, 2020—Lockdown extended until May 3, 2020 (Prime Minister’s Office, 2020)
	•May 1, 2020—Further lockdown extended for 2 weeks from May 4, 2020; however, variable activities across districts depending on current infection rates (Ministry of Home Affairs India, 2020a)
	•June 1, 2020—Still strict enforcement of lockdown in containment zones with re-opening of activities outside of containment zones (Ministry of Home Affairs India, 2020b)
	•26 June—Indian railways will not resume a regular service until at least 12 August due to a spike in cases (Associated Press, 2020)
Financial and socioeconomic activities	•The government in India announced US $2.1 billion aid to help the health sector fight the pandemic (Pai et al., 2020)
	•March 27, 2020—Government announces a $22.6 billion stimulus package to aid poorer communities affected by lockdown measures (Ahmed, 2020)
	•End March 2020—Interest rates reduced, Tata group has donated approximately $200 million and the Aditya Biria group approximately $70 million to fight the spread of the pandemic and provide medical supplies (Gandhi 2020)
	•May 13, 2020—Prime minister promises a $266 billion package to protect the economy from Covid-19 (Gupta & Sud, 2020)

Currently, there is not a cure for COVID-19; however, a number of medicines have been proposed and are undergoing trials (Sanders et al., 2020; Scavone et al., 2020). Recently, dexamethasone has been shown in the UK Recovery Trials to reduce deaths in ventilated patients and in those receiving oxygen only (Horby et al., 2020a). Remdesivir has shown encouraging results in one study after earlier concerns over an underpowered study (Beigel et al., 2020; ECDC, 2020; Wang et al., 2020). However a recent study among moderate patients failed to show similar benefit with remdesivir; consequently, still being considered as experimental (Dyer, 2020; McCreary and Angus, 2020; Spinner et al., 2020). Triple antiviral therapy is also showing promise in the management of COVID-19 patients although numbers are small (Hung et al., 2020); however, more studies are needed before any recommendations can be made. In addition, an appreciable number of vaccines are now in development (Checcucci et al., 2020; ECDC, 2020).

However, there is still considerable controversy surrounding the use of chloroquine and hydroxychloroquine with or without azithromycin for both prevention and treatment of COVID-19, following initial studies in China (Boulware et al., 2020; Cortegiani et al., 2020; Gao et al., 2020; Gautret et al., 2020). Internationally, concerns were raised about the lack of comparisons in the initial studies as well as potential harm including cardiac side-effects (Abena et al., 2020; Borba et al., 2020; Gautret et al., 2020; International Society of Antimicrobial Chemotherapy, 2020; ISAC/Elsevier, 2020; Ferner & Aronson, 2020; Littlejohn, 2020). Subsequent studies, including registry studies, have enhanced these concerns for hydroxychloroquine for both the prevention and treatment of COVID-19 (Boulware et al., 2020; Das et al., 2020; Geleris et al., 2020; Horby et al., 2020b; Littlejohn, 2020; Rosenberg et al., 2020). Consequently, the European Medicines Agency has advised against its prescribing outside of clinical trials (European Medicine Agency, 2020) and Das et al. (2020) in India also advised caution (Das et al., 2020). The study by Mehra et al. (2020) also showed increased mortality with chloroquine or hydroxychloroquine; however, this paper has now been retracted and is currently subject to external auditing (Editorial, 2020; Mehra et al., 2020a; Mehra et al., 2020b). In view of recent studies including the UK Recovery trial, the WHO has now halted the hydroxychloroquine arm in the ongoing Solidarity Trial and the National Institute of Health in the US has also halted the use of hydroxychloroquine in its studies (ECDC, 2020; Horby et al., 2020b; NIH, 2020; WHO, 2020c).

Having said this, the ICMR under the Ministry of Health and Family Welfare in India continues to recommend hydroxychloroquine for prophylaxis despite potential concerns (Kumar et al., 2020; Natnayas et al., 2020; Pulla, 2020; Rathi et al., 2020; Tilangi et al., 2020). In a recently published case controlled study, Chatterjee et al. (2020) demonstrated that the prescribing of four or more maintenance doses of hydroxychloroquine was associated with a significant reduction in the odds of healthcare workers getting COVID-19 (Chatterjee et al., 2020). Other treatments initially recommended by ICMR and others include lopinavir-ritonavir, although there is contrasting data regarding its effectiveness in COVID-19 patients (Bhatnagar et al., 2020; Cao et al., 2020; Kumar et al., 2020). More recently, the WHO has discontinued the lopinavir-ritonavir arm of the Solidarity trial with the interim results showing this combination demonstrated little or no reduction in the mortality of hospitalized COVID-19 patients when compared to standard of care (WHO, 2020c). Overall, further studies are needed before specific treatments can be robustly recommended given the continuing concerns with a number of treatments, the redaction of recent studies and issues with study design and setting (Bae et al., 2020; Godman, 2020; International Society of Antimicrobial Chemotherapy, 2020; Mehra et al., 2020b).

We are aware the endorsement of hydroxychloroquine has resulted in appreciably increased use in a number countries and localities along with increased prices, although there have been hospitalisations and deaths from poisoning (Abena et al., 2020; Buasi and Adebayo, 2020; Haque et al., 2020; Kwasi, 2020; Mendel et al., 2020; Salo, 2020; Topp, 2020; Vaduganathan et al., 2020). Increased prices can be a concern in India with potentially detrimental consequences for families if limited available funds for priority disease areas including NCDs are being diverted towards purchasing treatments where there are ongoing controversy along with purchasing of PPE at increased prices (Business Today, 2020).

### Study Aims and Objectives

In view of the current situation in India, we believed there was an urgent need to assess the impact of COVID-19 on the availability and prices of medicines and other technologies to prevent and treat COVID-19 in community pharmacies. The key considerations are the high rates of both infectious and non-infectious diseases in India, issues with sanitation and crowded streets, the catastrophic consequences for families when members become ill, and fears regarding the availability of regular medicines during the pandemic (Kastor & Mohanty, 2018; Selvaraj et al., 2018; Statista, 2020). Community pharmacists are a particular target since they currently play a key role in the management of diseases in India, with ongoing issues with access to physicians and high co-payments especially in rural areas (Abdulsalim et al., 2018; Daftary et al., 2019; Nafade et al., 2019). They are also in a good position to suggest to patients with more severe symptoms that they seek additional help (Amariles et al., 2020). This is important since it is known it can be difficult to differentiate respiratory tract infections from COVID-19 in patients presenting with coughs and fever (Ongole et al., 2020).

We have seen prices rise for essential medicines in LMICs over time and for treatments for COVID-19, as well as appreciable price increases for the materials to make PPE in India following the start of the pandemic. In view of this, we believe it is important to evaluate the current situation in India regarding pertinent medicines and PPE to provide future guidance at this critical time (Kasonde et al., 2019; Buasi & Adebayo, 2020; Business Today, 2020). Consequently, we sought to address this through direct contact with community pharmacists across India. However, we are aware of government regulations regarding the pricing of essential medicines in India (HealthWorld, 2016; Pavithra, 2019). As a result, in this initial study we sought to assess the utilisation, availability and price changes of medicines and PPE for COVID-19 among a number of cities across India to provide future direction to key stakeholder groups in India. Senior level co-authors would also be targeted to provide guidance based on their experiences across LMICs.

We have not assessed the influence of the various Government initiatives (Table 1) on the prevalence and mortality of COVID-19 in India. We are aware of the concerns about the testing rates for COVID-19 certainly initially as well as evolving strategies to address ongoing spikes in infection rates as lockdown measures are eased and with the advent of the monsoons (Associated Press, 2020; Siddiqui, 2020). We will though be monitoring morbidity and mortality rates alongside the unintended consequences of lockdown and other measures given rising prevalence rates of COVID-19 in India and existing high prevalence rates for both infectious and non-infectious diseases, and will be reporting on this in future studies. Unintended consequences include issues of increased gender violence as well as mental health issues associated with any stigma with COVID-19 as well as lockdown measures (IFRC & WHO, 2020; Rajkumar, 2020; SADC, 2020). In addition, rising rates of NCDs especially if patients cannot attend clinics or receive their medicines due to lockdown measures as well as having affordability issues with medicines through lack of earnings due to lockdown and other measures (Basu, 2020; Kluge et al., 2020).

## Methodology

We initially undertook a narrative review of the current situation regarding COVID-19 in India and on suggested treatments. We subsequently undertook quantitative research in the form of a survey. The narrative review included a review of current and proposed treatment approaches including vaccines and recommendations for preventing and managing COVID-19, including the role of community pharmacists as well as issues of misinformation. We did not systematically review the papers or other information sources for their quality using well-known scales such as the Newcastle-Ottawa scale as some of the papers quoted are in pre-publication format and we have used a considerable number of internet sources (Almeida et al., 2018). However, the publications and internet sources were filtered by the co-authors to add robustness to the paper and its suggestions.

The information sourced from the pragmatic review of the literature was combined with a questionnaire survey among community pharmacies (**Appendix 1**) to assess the situation regarding prices, availability and usage patterns of carefully selected medicines that could potentially be used in the management of COVID-19, as well as PPE, soon after the start of the pandemic.

For this rapid analysis, we selected via purposive sampling representation of pharmacies from across India. This included Ahmedabad, Jodhpur, and Pune as part of Western India, West Singhbhum and Kolkata as part of Eastern India, and Delhi in northern India. Convenience sampling in these cities was used to select pharmacists through emails, telephone contact, personal contacts and other mechanisms. There was no sample size calculation as there was no previous data in India to base calculations upon. However, the intention was to undertake the research among an appreciable number of community pharmacies across India to gain good insights and provide a basis for future studies if needed.

Key questions were to assess patterns of demand, availability, and price changes of selected medicines and equipment, as well as the potential future role of pharmacists to reduce misinformation. These are contained in Box 2 (building on Appendix 1). Those conducting the research were provided with an Excel spreadsheet of the questions to complete. The questions were open ended as we were aware that in a number of situations we would be unable to obtain exact details of changes in utilisation patterns and prices; however, we wanted to capture data including more general information for this initial study. The answers were collated where possible into logical bands for comparisons with other countries such as Bangladesh (Haque et al., 2020). These bands were not pre-defined as this was an exploratory rapid pilot study, with changes in prices based on local prices. In addition, general information would be sufficient if the pharmacists were unable to be specific given the exploratory nature of this study.

Box 2 **Open ended questions to community pharmacists in India regarding pertinent medicines and equipment to prevent and treat COVID-19.**

- Geographic location (Region)
- What changes in medicine purchasing patterns have you noticed in your pharmacy from the beginning of March 2020 until the end of May 2020 for antimalarials (hydroxychloroquine), antibiotics (e.g. azithromycin and co-amoxiclav), multivitamins including Vitamin C and analgesics based on invoices where possible or other information sources; alternatively, impressions. Similarly for any shortages of these medicines during the same period
- What changes in medicine prices have you noticed for targeted medicines from the beginning of March 2020 until end May 2020 for antimalarials, antibiotics, multivitamins and analgesics (based on invoices or other information sources where possible)
- Similarly, for PPE (utilisation, prices and shortages) - beginning of March 2020 until the end May 2020 - based on invoices or other information sources/impressions
- Any suggestions for addressing inappropriate self-medication for the future including addressing misinformation and its potential catastrophic consequences for patients?

**FIGURE 1:**
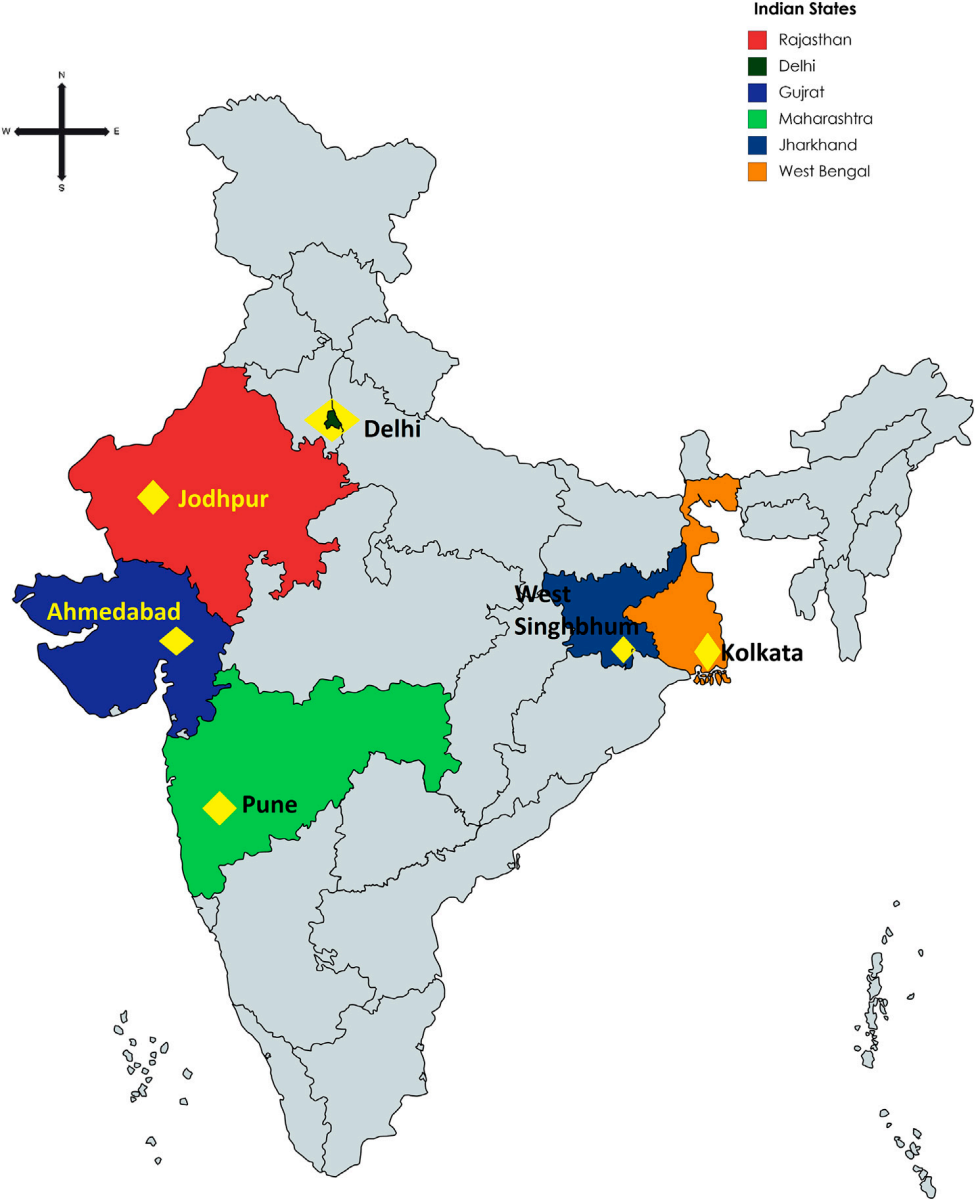
Location of participating pharmacies in India.

The pharmacists were briefed on the objectives of the study with the option to participate or not, with confidentiality maintained throughout. Our hypothesis, based on findings in other countries, was that there would be shortages of some of the medicines, although countered in India as a chief producer of medicines with export bans in place certainly initially (Duffy & Hussain, 2020) Price rises especially for medicines potentially tempered though by the Ministry of Pharma and Consumer Affairs being instructed to take necessary action to regulate these for PPE and other health related materials alongside existing regulations for (Table 1) (WHO India, 2020e). The findings were compiled into a tabular format. No formal statistical analysis was performed as the level of detail varied considerably across the pharmacies.

We subsequently combined the data collected using the experience of the co-authors regarding key issues including pharmaceutical care, health policy and self-purchasing in LMICs to provide future direction, building on comments from the interviewed pharmacists. We have previously successfully used this approach to provide future direction in LMICs (Godman et al., 2018; Godman et al., 2019a; Godman et al., 2020a; Godman et al., 2020b; Godman et al., 2020c; Godman et al., 2020d).

Ethical approval for this study was not required according to national legislation and institutional guidelines. However, all pharmacists freely provided the requested information having been given the opportunity to refuse to participate if wished. This is in line with previous studies undertaken by the co-authors in related areas including analysis of policies to enhance the rationale use of medicines and biosimilars, pricing policies and issues surrounding generics, which involved direct contact with health authority personnel and other key stakeholders (Moorkens et al., 2017; Godman et al., 2019b; Gad et al., 2020; Godman et al., 2020a; Godman et al., 2020c; Miljković et al., 2020).

## Results

Overall, 135 pharmacies were visited to give a response rate of 82% (Table 2).

We first report on changes in utilisation patterns before reporting on any price changes seen with the various medicines and equipment as well as any shortages. The reported figures are absolute figures based on interviewee feedback.

**TABLE 2:** Details of responses among pharmacy/drugs stores approached. NB—Groups 1 and 2 refer to the consolidation co-ordination of the findings by the principal co-author involved. The pharmacies were located throughout India (Figure 1; Table 3).

	How many pharmacies approached (in number)	How many accepted to take part (in number)	How many refused (in number)
Group 1	69	56	13
Group 2	66	55	11
Total (total and %)	135	111 (82%)	24 (18%)

**TABLE 3:** Community Pharmacy location in India.

Location and State	Number of pharmacies where data was collected
JODHPUR, Rajasthan	44
Jharkhand	12
Delhi, union territory	15
Ahmedabad, Gujarat	15
Kolkata, West Bengal	15
Pune, Maharashtra	10
Total	111

### Utilisation

Table 4 depicts changes in utilisation patterns for the various medicines, vitamins and PPE equipment and hand sanitisers from the beginning of March until the end of May 2020. Encouragingly, no change or decreased utilisation of antimalarials was seen in 45% of pharmacies taking part, with similarly limited increases in the utilisation of antibiotics (42.3% of pharmacies taking part). An appreciable increase in antibiotic utilisation was seen though in a small number of pharmacies.

**TABLE 4:** Utilisation changes for medicines and PPE between beginning March 2020 and end May 2020 among 111 pharmacies in India.

	Antimalarials	Antibiotics	Analgesics	Vitamins/immune boosters	Face masks/PPE
Decrease (including due to prescription required and no prescription)	25	23	21	1	0
No change	25	41	15	10	2
Slight increase	10	5	1	2	0
Increase (not specified)	49	30	62	79	54
1.5 to 2 fold increase	1	3	0	2	0
2 to 3 fold increae	0	2	3	9	3
4 to 5 fold increase	1	1	6	0	3
Over 5 fold increase	0	6	3	8	49
Total	111	111	111	111	111
No change/decrease	50	64	36	11	2
% of the total	45.0	57.7	32.4	9.9	1.8
Increase %	55.0	42.3	67.6	90.1	98.2

NB: No change also includes situations where not dispensed without a prescription (antimalarials and antibiotics).

Encouragingly, there was an increase in the purchasing of PPE and vitamins to boost the immune system in over 90% of pharmacies taking part, nearing 100% for PPE. It is likely that we will continue to see such increases in the purchasing of both of these if COVID-19 infection rates continue to increase following any easing of lockdown measures.

### Price Changes

Table 5 depicts price changes for pertinent medicines and PPE. Encouragingly, there were limited price changes for antimalarials (only 16.2% of the pharmacies) and antibiotics (8.1% of the pharmacies). Contrasting with this, price increases were seen for PPE among over 90% of the pharmacies taking part in the study. This was only a slight increase, or an increase in only one of the three months, in 39.6% of pharmacies taking part.

**TABLE 5:** Price changes for medicines and PPE between beginning March 2020 and end May 2020 among 111 pharmacies in India.

	Antimalarials	Antibiotics	Analgesics	Vitamins/immune boosters	Face masks/PPE
Decrease	0	0	1	0	0
No change	93	102	84	51	10
Slight increase (including only one month out of the 3)	10	2	2	2	44
Increase (not specified)	2	3	19	49	52
Up to 2 fold	0	0	0	0	0
2 to 3 fold increase	1	4	4	4	1
3 to 4 fold increase	0	0	1	2	1
4 to 5 fold increase	2	0	0	3	2
Over 5 fold increase	3	0	0	0	1
Total	111	111	111	111	111
No change/decrease	93	102	85	51	10
% of the total	83.8	91.9	76.6	45.9	9.0
Increase %	16.2	8.1	23.4	54.1	91.0

### Medicine and Prevention Shortages

Perhaps not surprisingly, shortages of some medicines and PPE were seen among the pharmacies in India (Table 6). These were principally confined to antimalarial medicines (70.3%) and PPE (88.3%).

**TABLE 6:** Shortages for medicines and PPE between beginning March 2020 and end May 2020 among 111 pharmacies in India.

	Antimalarials	Antibiotics	Analgesics	Vitamins/immune boosters	Face masks/PPE
No change/available	33	100	109	84	13
Part shortages (1-2 months	25	9	0	2	43
Shortages (unspecified)	53	2	2	2	55
Total	111	111	111	111	111
No change % of total	29.7	90.1	98.2	75.7	11.7
Shortages %	70.3	9.9	1.8	24.3	88.3

### Potential Ways Forward to Address Misinformation and Enhance Appropriate Use of Medicines and Equipment Across Sectors

Possible strategies based on interviewee and co-author feedback to address misinformation and other concerns regarding the management of COVID-19 and any unintended consequences are included in Table 7. We believe this is especially important for patients with NCDs given rising rates in India, and we will be researching this further.

**TABLE 7:** Key activities among stakeholder groups to improve prevention and management of patients with COVID-19.

Stakeholder group	Suggested activities
Government	•Encourage an evidence-based environment for key recommendations and pronouncements especially when proposing activities to prevent and treat COVID-19. This is particularly important given the controversies that continue to surround hydroxychloroquine and family resources spent on recommended treatments for the management of COVID-19 will mean less monies for other medicines and food especially among patients with existing diseases including chronic NCDs (India State-Level Disease Burden Initiative Diabetes C, 2018; Ramakrishnan et al., 2019). This is in line with advice and recommendations from the council for international organisations of medical sciences (Council for International Organizations of Medical Sciences, 2020)
	•Ensure that there is active dissemination of suggested activities to prevent the spread of COVID-19 including sanitation in social media and other platforms given the increasing impact of social media in LMICs (Soon, 2020); however, mindful of the likely situation regarding concerns with social distancing and lack of clean water in many households
	•Keep track of misinformation and any false claims for medicines or technologies for the diagnosis and management of COVID-19 given ongoing controversies regarding some recommended treatments and the financial consequences involved if scarce resources are diverted to purchase these products
	•Ensure swift and strict action against fraudulent advertisements and sale of unproven products in this time of emergency building on activities in other countries (Davis, 2020; Dzirutwe, 2020)
	•Continue with planned programmes to improve the management of patients with chronic NCDs, reduce AMR and actively treat infectious diseases to minimise the unintended consequences of COVID-19
	•Continue to enhance local production of PPE where pertinent to address current shortages as well as seek mechanisms to keep any prices rises to a minimum (Bhattacharya et al., 2020; Pandey, 2020)
	•Continue to adopt a phased approach to the easing of lockdown measures, with rapid re-introduction if needed (Habersaat et al., 2020)
Physicians	•Enhance a philosophy of evidence-based medicine starting in medical school and continuing post qualification building on guideline initiatives in India (Mehndiratta et al., 2017), given current controversies surrounding hydroxychloroquine, lopinavir- ritonavir and remdesivir
	•As part of this, continue to ensure prescribed/recommended treatments are evidence based through postgraduate training and other continuous professional development activities post qualification
	•Continue to encourage where possible appropriate management of chronic NCDs including adherence to prescribed medicines giving rising rates of NCDs in India
Pharmacists	•Continue to encourage, where possible, the adoption of recommended strategies to prevent the spread of COVID-19 including pertinent PPE. This includes hand sanitisation as well as appropriate wearing of face masks
	•Try to ensure that PPE and medicines, or suitable alternatives, helpful for patients with COVID-19 are routinely available, and help ensure where possible that any price rises are kept to a minimum given the number of people in India going into poverty when family members become ill—helped by current legislation regarding price rises especially for essential medicines coupled with ongoing programmes promoting generics (HealthWorld, 2016; Pavithra, 2019)
	•Continue to argue against the need for antibiotics where this is a concern - helped recently by increasing recognition of the need for a prescription before dispensing (similar to antimalarial medicines)
	•Work with patients to enhance good adherence to purchased medicines including those for NCDs and encourage appropriate referrals to other professionals where pertinent especially for moderate to severe disease
	•Potentially become involved in vaccination programmes where there is unmet need as published studies suggest that when pharmacists provide immunizations they substantially increase the number of vaccinated people which is important at this time (Ogunleye et al., 2020)
Patients/Patient organisations	•Where possible, engage with social media and channels to help promote evidence-based approaches to the prevention and management of COVID-19 given the level of misinformation seen to date—ensure messages are as clear as possible and in a positive language (Habersaat et al., 2020)
	•Monitor the spread of misinformation regarding the treatment of COVID-19 with fake or unproven medical products and procedures that claim to diagnose, prevent or cure COVID-19, and work with governments and other key stakeholder groups to minimise their impact (Habersaat et al., 2020)
	•Work with governments and other bodies to minimise the potential consequences of COVID-19 on mental health issues for healthcare professionals and patients
	•Work with patients and groups to reduce concerns with stigma and reassure COVID-19 patients that they should pose no threat to the community after treatment and continue with their lives
	•Seek to educate patients/build on current programmes regarding self-management in patients with chronic NCDs including the priority for medication adherence given lockdown restrictions
	•Help explore new technologies with key stakeholders including the growing use of telemedicine approaches and other approaches to reduce the reliance on clinic attendance to manage NCDs

NB: LMICs, low- and middle-income countries; NCDs, non-communicable diseases; PPE, personal protective equipment.

## Discussion

We believe this is one of the first comprehensive studies worldwide to assess the impact of COVID-19 on the utilisation, availability and price changes of pertinent medicines and PPE used to prevent and treat COVID-19 among LMICs with a high response rate (82%) among those taking part. In India, high patient co-payments have contributed to potentially catastrophic consequences for families when members become ill, although this is beginning to change helped by ongoing legislation to moderate prices of essential medicines (Kastor and Mohanty, 2018; Reddy, 2018; Pavithra, 2019; Sood and Wagner, 2020).

As expected, our study demonstrates an increase in the utilisation of a number of suggested medicines and PPE, enhanced by endorsement of certain approaches including increased protection with PPE (Table 4). However, increases in utilisation were not as great for antimalarial treatments (55% of pharmacies noting an increase) and antibiotics (42.3% of pharmacies noting an increase) compared with analgesics (67.6%) and vitamins (90.1%). This is in line initiatives by the government advising pharmacists not to sell antimalarials and antibiotics without a prescription. The utilisation patterns seen in India appear similar to the situation in Bangladesh with antimalarial tablets (48.8% of pharmacies and drug stores seeing an increase), analgesics (97.6% of pharmacies and drug stores seeing an increase) and vitamins (90.6% seeing an increase), but lower than in Bangladesh with respect to antibiotics (with 70.6% of stores noting an increase), perhaps reflecting greater scrutiny over the need for patients to have a prescription for an antibiotic in India (Haque et al., 2020).

Price increases were also seen for pertinent medicines (Table 5), which was expected given some of the shortages seen (Table 6). However, price increases appeared appreciably lower than seen in Bangladesh with only 16.2% of pharmacies in India reporting a price increase for antimalarials vs. 50% in Bangladesh and only 8.1% of pharmacies in India reporting price increases for antibiotics vs. 34.7% in Bangladesh (Haque et al., 2020). We believe this reflects greater price controls for medicines in India compared with Bangladesh, providing direction to countries such as Bangladesh during the current pandemic and after (HealthWorld, 2016; Pavithra, 2019). A lower number of pharmacies in India (23.4%) also reported price increases for analgesics vs. 45.3% in Bangladesh (Haque et al., 2020). This may reflect greater production of ingredients for medicines (API) in India than seen in Bangladesh coupled with Government price controls (Pavithra, 2019). However, further research will be needed before any definitive statement can be made especially as shortages of antimalarials were seen in India during the study period (70.3% of pharmacies), although this was only short term in a third of these (Table 6). Shortages of antimalarial medicines were seen in 54.1% of pharmacies and drug stores in Bangladesh during the same period (Haque et al., 2020).

As mentioned, we observed an appreciable increase in the use of PPE in both Bangladesh (over 95% of pharmacies and drug stores respectively) and India (98.2% respectively) during the study period (Haque et al., 2020). This was typically accompanied by shortages and price rises in both countries, some of which were substantial (Haque et al., 2020). We have also seen substantial increase in the price of PPE in other LMICs as the pandemic progresses (Weston, 2020).

Table 7 highlights potential activities that can be undertaken by key stakeholder groups in India and more widely to address issues and concerns with COVID-19. This includes addressing the unintended consequences of COVID-19 including issues of stigma as well as addressing concerns with appropriate identification and management of other infectious diseases apart from COVID-19 as well as among patients with chronic NCDs given rising rates of CVD and diabetes in India. We will be monitoring the situation with respect to hydroxychloroquine given ongoing controversies across countries.

We are aware of a number of limitations with this study. These include the fact that we were unable to obtain exact details on changes in the utilisation and prices of pertinent medicines and PPEs from all the pharmacists visited due to issues of confidentiality and having the data readily to hand. We also did not cover all the regions in India. In addition, we did not break down antibiotics into azithromycin and other antibiotics such as amoxycillin in view of the study constraints. We also did not ask about shortages of other medicines such as those to treat patients with NCDs. Alongside this, we are aware that some of the shortages initially may have been due to transport and logistic issues. A number of these issues will be addressed in future studies. However, we are confident our findings can be helpful for future planning purposes in India and wider given the number of community pharmacies that were involved.

## Conclusion

In our study we have seen increases in utilisation and prices as well as shortages of pertinent medicines and PPE used to prevent and treat COVID-19 in India. Encouragingly, the extent of shortages and price increases in India were not as high as originally expected, potentially helped by local production of medicines, including the active ingredients, greater scrutiny over the dispensing of antimalarials and antibiotics without a prescription, as well as government control over prices including recently; with similarities with Bangladesh. There are ongoing concerns and challenges regarding potential treatments including antimalarials, which needs urgent addressing. Increasing recognition of the need for evidence-based medicine in terms of treatment recommendations and prescribing can help further reduce inappropriate treatments being recommended, prescribed or dispensed. Patient organisations can also play a role through social media and other platforms to reduce the extent of misinformation and its potential damaging consequences. We will be monitoring this in the future.

## Data Availability

The raw data supporting the conclusions of this article will be made available by the authors, without undue reservation, to any qualified researcher.

## Appendix 1—Questionnaire to community pharmacists in India.

The Questionnaire is designed to ascertain changes in the utilisation, prices and shortages of possible medicines and equipment to prevent and treat patients with COVID-19. You are free to participate or not, and confidentiality will be maintained.

Question 1: What is your location (City and region)?

Question 2: What changes in medicine purchasing patterns have you noticed in your pharmacy from the beginning of March, i.e. soon after the start of the pandemic but before major travel and other restrictions until the end of May, for antimalarials (hydroxychloroquine), antibiotics (e.g. azithromycin and co-amoxiclav), multivitamins including Vitamin C and analgesics [Please note - This is based on invoices where possible from prior to the beginning of March or other information sources; alternatively impressions (free text)]

Question 3: What changes in prices have you noticed in your pharmacy from the beginning of March until the end of May, for antimalarials (hydroxychloroquine), antibiotics (e.g. azithromycin and co-amoxiclav), multivitamins including Vitamin C and analgesics [Please note - This is based on invoices where possible from prior to the beginning of March or other information sources; alternatively impressions (free text)]

Question 4: Have there been any shortages for antimalarials (hydroxychloroquine), antibiotics (e.g. azithromycin and co-amoxiclav), multivitamins including Vitamin C or analgesics in your pharmacy from the beginning of March until the end of May, for antimalarials (hydroxychloroquine), antibiotics (e.g. azithromycin and co-amoxiclav), multivitamins including Vitamin C and analgesics? If so, what is the extent of any shortages (free text)?

Question 5: What has been the changes in utilisation, prices and potential shortages for personal protection equipment (PPE) such as hand sanitisers and face masks in your pharmacy from the beginning of March until the end of May? [[Please note - This is based on invoices where possible from prior to the beginning of March or other information sources; alternatively impressions (free text)]

Question 6: What suggestions do you have for the authorities in India to reduce misinformation regarding different management and treatment approaches for new pandemics as well as reduce inappropriate self-medication with antimicrobials (free text)?
