# Supplementary material for: Utilisation, Availability and Price Changes of Medicines and Protection Equipment for COVID-19 Among Selected Regions in India: Findings and Implications
Source: Front Pharmacol. 2021 Jan 14;11:582154. doi: 10.3389/fphar.2020.582154 (PMC7898674; doi:10.3389/fphar.2020.582154)
Supplement: Supplementary file 1 [file table1.docx]

**Appendix 1 – Questionnaire to community pharmacists in India**

The Questionnaire is designed to ascertain changes in the utilisation, prices and shortages of possible medicines and equipment to prevent and treat patients with COVID-19. You are free to participate or not, and confidentiality will be maintained.

Question 1: What is your location (City and region)

Question 2: What changes in medicine purchasing patterns have you noticed in your pharmacy from the beginning of March, i.e. soon after the start of the pandemic but before major travel and other restrictions until the end of May, for antimalarials (hydroxychloroquine), antibiotics (e.g. azithromycin and co-amoxiclav), multivitamins including Vitamin C and analgesics. This based on invoices where possible from prior to the beginning of March or other information sources; alternatively impressions (free text)

Question 3: What changes in prices have you noticed in your pharmacy from the beginning of March until the end of May, for antimalarials (hydroxychloroquine), antibiotics (e.g. azithromycin and co-amoxiclav), multivitamins including Vitamin C and analgesics. This based on invoices where possible from prior to the beginning of March or other information sources; alternatively impressions (free text)

Question 4: Have there been any shortages for antimalarials (hydroxychloroquine), antibiotics (e.g. azithromycin and co-amoxiclav), multivitamins including Vitamin C or analgesics in your pharmacy from the beginning of March until the end of May, for antimalarials (hydroxychloroquine), antibiotics (e.g. azithromycin and co-amoxiclav), multivitamins including Vitamin C and analgesics. If so, what is the extent of any shortages (free text)

Question 5: What has been the changes in utilisation, prices and potential shortages for personal protection equipment (PPE) such as hand sanitisers and face masks in your pharmacy from the beginning of March until the end of May. This based on invoices where possible from prior to the beginning of March or other information sources; alternatively impressions (free text)

Question 6: What suggestions do you have for the authorities in India to reduce misinformation regarding different management and treatment approaches for new pandemics as well as reduce inappropriate self-medication with antimicrobials (free text)?
